# Supplementary figures and images for: Proteomic and evolutionary analyses of sperm activation identify uncharacterized genes in Caenorhabditis nematodes
Source: BMC Genomics. 2018 Aug 7;19:593. doi: 10.1186/s12864-018-4980-7 (PMC6081950; doi:10.1186/s12864-018-4980-7)

# Unactivated Spermatid: Mean Relative Abundance

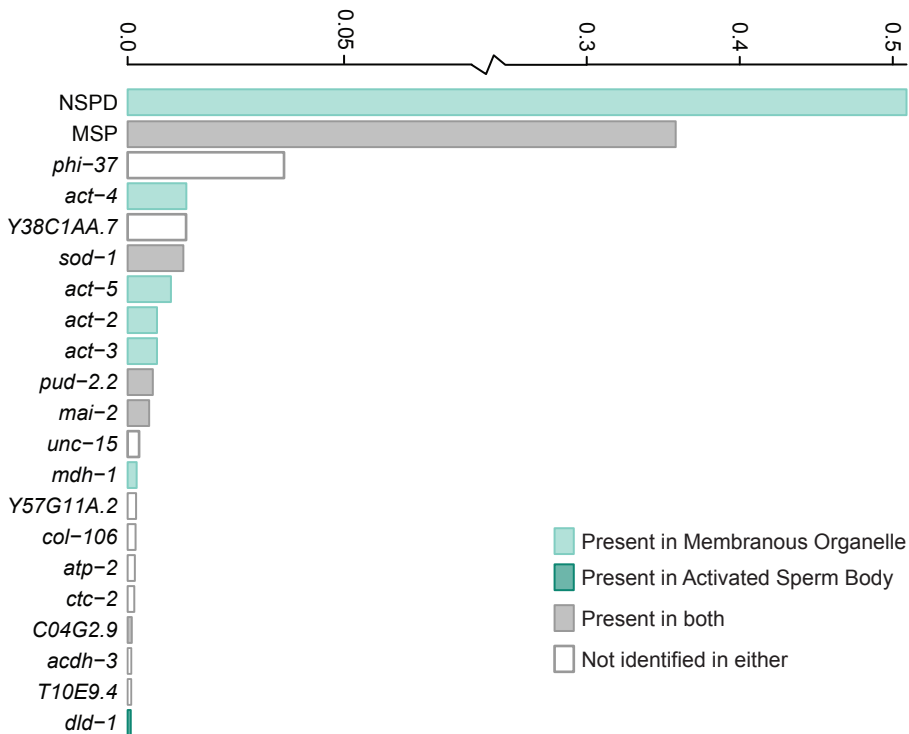

Supplement: Supplementary file 1 — The un-activated sperm proteome of C. elegans. The majority of the proteome is comprised of the Nematode-Specific Peptide family, group D (NSPD) and the Major Sperm Protein (MSP). Protein abundance is shown as the relative mean normalized spectrum abundance frequency. Proteins found to be unique to either the membranous organelle or activated sperm proteomes are highlighted in teal, while proteins found in both proteomes are shown in gray. Proteins shown in white were not identified in the membranous organelle or activated sperm proteomes, but were found in the previously published un-activated spermatid proteome of Ma et al. [21]. (PDF 863 kb) [file 12864_2018_4980_MOESM1_ESM.pdf]

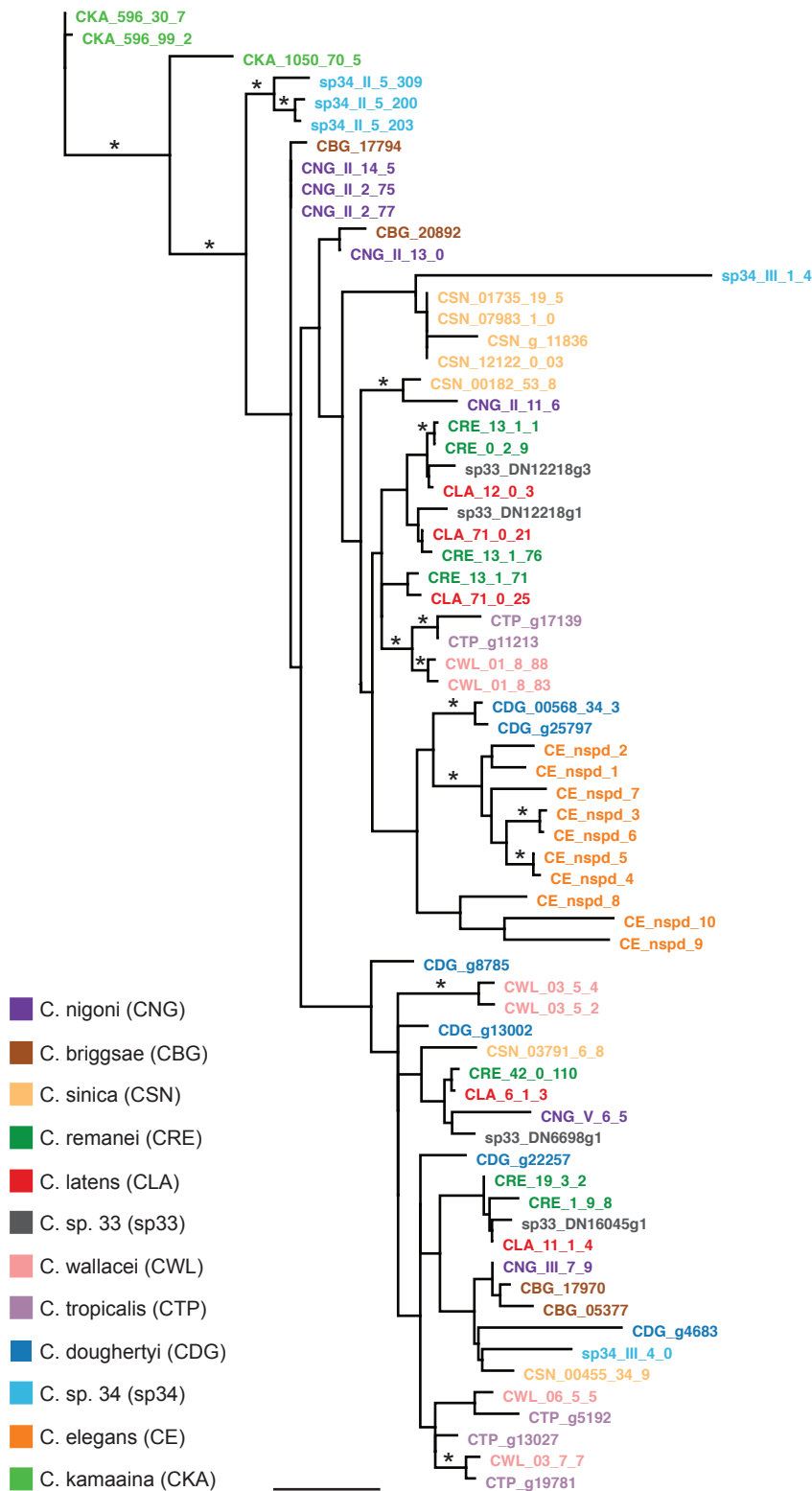

0.2

Supplement: Supplementary file 5 — An unrooted maximum likelihood phylogeny for the Nematode-Specific Peptide family, group D (NSPD). Genes tend to cluster within species and do not recapitulate an evolutionary history of gene orthology. Asterisks denote bootstrap values greater than 80%. (PDF 945 kb) [file 12864_2018_4980_MOESM5_ESM.pdf]

NSPF-1 Orthologous Genes

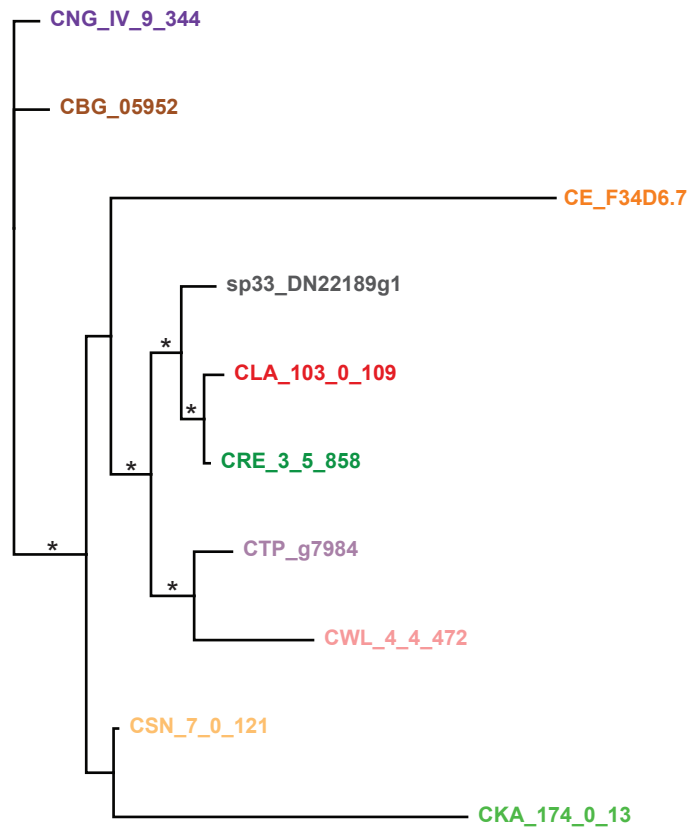

0.09

NSPF-3 Orthologous Genes

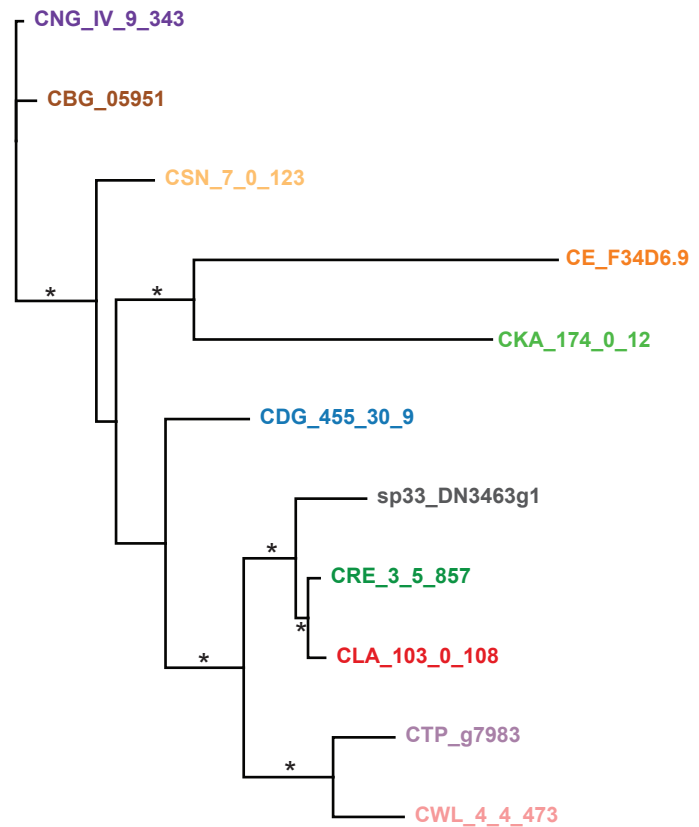

0.08

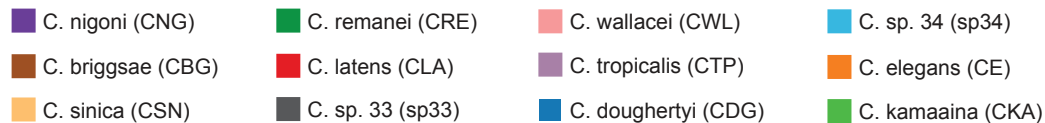

Supplement: Supplementary file 7 — Unrooted maximum likelihood phylogenies for the Nematode-Specific Peptide family, group F (NSPF) orthologous genes. Overall, gene trees recapitulate species relationships. Asterisks denote bootstrap values greater than 80%. (PDF 867 kb) [file 12864_2018_4980_MOESM7_ESM.pdf]
