# Supplementary material for: Proteomic and evolutionary analyses of sperm activation identify uncharacterized genes in Caenorhabditis nematodes
Source: BMC Genomics. 2018 Aug 7;19:593. doi: 10.1186/s12864-018-4980-7 (PMC6081950; doi:10.1186/s12864-018-4980-7)
Supplement: Supplementary file 3 — Gene annotations for the NSPD and NSPF gene families. Orthologous genes for the Nematode-Specific Peptide family, group D (NSPD) and Nematode-Specific Peptide family, group F (NSPF) family in 11 Caenorhabditis species. Annotations are listed by species, along with the gene start position and coding sequence length. (DOCX 29 kb) [file 12864_2018_4980_MOESM3_ESM.docx]

**Additional file 3A.** Annotated Nematode-Specific Peptide, Family D (NSPD) orthologs in 11 *Caenorhabditis* species. Gene identifiers are comprised of the species code, scaffold or chromosome and the first three digits of the sequence position. If the gene was previously annotated, the species code and gene ID number are given. The transcript number is given for *C. sp. 33*. The gene start position and coding sequence length (CDS) are given.

| **Species** | **Orthologous Gene** | **Position** | **CDS (bp)** |
| --- | --- | --- | --- |
| *C. kamaaina* | CKA_596_30_7 | 596: 30,747 | 213 |
|  | CKA_596_99_2 | 569: 99,272 | 213 |
|  | CKA_1050_70_5 | 1050: 70,532 | 207 |
| *C. elegans* | *nspd-1* | I: 6,097,496 | 213 |
|  | *nspd-2* | IV: 9,844,029 | 213 |
|  | *nspd-3* | IV: 5,162,743 | 216 |
|  | *nspd-4* | II: 4,842,564 | 216 |
|  | *nspd-5* | II: 4,860,063 | 216 |
|  | *nspd-6* | IV: 5,165,420 | 216 |
|  | *nspd-7* | IV: 10,268,662 | 213 |
|  | *nspd-8* | I: 2,551,375 | 216 |
|  | *nspd-9* | III: 4,830,010 | 216 |
|  | *nspd-10* | I: 10,878,047 | 300 |
| *C. sp. 34* | sp34_II_5_200 | II: 5,200,946 | 204 |
|  | sp34_II_5_203 | II: 5,203,656 | 204 |
|  | sp34_II_5_309 | II: 5,309,896 | 204 |
|  | sp34_III_1_4 | III: 1,428,443 | 186 |
|  | sp34_III_4_0 | III: 4,066,062 | 201 |
| *C. doughertyi* | CDG_g13002 | 00030: 189,285 | 222 |
|  | CDG_g22257 | 00325: 51,418 | 219 |
|  | CDG_g25797 | 00530: 19,146 | 219 |
|  | CDG_00568_34_3 | 00568: 34,393 | 219 |
|  | CDG_g4683 | 00965: 33,956 | 225 |
|  | CDG_g8785 | 01780: 7,038 | 225 |
| *C. tropicalis* | CTP_g5192 | 593: 61,448 | 219 |
|  | CTP_g11213 | 629: 13,905,030 | 219 |
|  | CTP_g13027 | 629: 19,635,262 | 219 |
|  | CTP_g17139 | 630: 1,632,560 | 219 |
|  | CTP_g19781 | 630: 11,802,730 | 219 |
| *C. wallacei* | CWL_01_8_83 | 01: 8,834,137 | 219 |
|  | CWL_01_8_88 | 01: 8,878,264 | 219 |
|  | CWL_03_5_2 | 03: 5,254,378 | 213 |
|  | CWL_03_5_4 | 03: 5,471,220 | 219 |
|  | CWL_03_7_7 | 03: 7,784,950 | 216 |
|  | CWL_06_5_5 | 06: 5,522,008 | 213 |
| *C. sp. 33* | sp33_DN12218 | 303-85 | 219 |
|  | sp33_DN16045 | 304-83 | 222 |
|  | sp33_DN6698 | 322-101 | 222 |
|  | sp33_DN12218 | 326-108 | 219 |

Additional file 3A *continued*

| **Species** | **Orthologous Gene** | | **Position** | | **CDS (bp)** | |
| --- | --- | --- | --- | --- | --- | --- |
| *C. latens* | | CLA_6_1_3 | | 6: 1,302,855 | | 213 |
|  |  | CLA_11_1_4 | | 11: 1,462,083 | | 216 |
|  |  | CLA_12_0_3 | | 12: 349,321 | | 219 |
|  |  | CLA_71_0_21 | | 71: 211,582 | | 219 |
|  |  | CLA_71_0_25 | | 71: 257,332 | | 219 |
| *C. remanei* | | CRE_0_2_9 | | 0: 2,951,349 | | 219 |
|  |  | CRE_1_9_8 | | 1: 9,876,147 | | 294 |
|  |  | CRE_13_1_1 | | 13: 1,117,735 | | 219 |
|  |  | CRE_13_1_71 | | 13: 1,719,168 | | 219 |
|  |  | CRE_13_1_76 | | 13: 1,763,196 | | 219 |
|  |  | CRE_19_3_2 | | 19: 321,459 | | 216 |
|  |  | CRE_42_0_110 | | 42: 11,077 | | 213 |
| *C. sinica* | CSN_00182_53_8 | | 00182: 53,813 | | 222 | |
|  | CSN_00455_34_9 | | 00455: 34,939 | | 219 | |
|  | CSN_g11836 | | 00474: 6,362 | | 540 | |
|  | CSN_01735_19_5 | | 01735: 19,521 | | 219 | |
|  | CSN_03791_6_8 | | 03791: 6,885 | | 231 | |
|  | CSN_07983_1_0 | | 07983: 1,035 | | 219 | |
|  | CSN_12122_0_3 | | 12122: 314 | | 219 | |
| *C. briggsae* | CBG_20892 | | II: 11,845,722 | | 219 | |
|  | CBG_17794 | | II: 13,221,469 | | 216 | |
|  | CBG_17970 | | III: 7,245,342 | | 216 | |
|  | CBG_05377 | | IV: 7,190,570 | | 300 | |
| *C. nigoni* | CNG_II_2_75 | | II: 2,751,887 | | 216 | |
|  | CNG_II_2_77 | | II: 2,770,958 | | 216 | |
|  | CNG_II_11_6 | | II: 11,674,034 | | 213 | |
|  | CNG_II_13_0 | | II: 13,024,658 | | 216 | |
|  | CNG_II_14_5 | | II: 14,564,036 | | 216 | |
|  | CNG_III_7_9 | | III: 7,950,129 | | 216 | |
|  | CNG_V_6_5 | | V: 6,586,997 | | 213 | |

**Additional file 3B.** Annotated Nematode-Specific Peptide family, group F (NSPF) orthologs in 11 *Caenorhabditis* species. Gene identifiers are comprised of the species code, scaffold or chromosome and the first three digits of the sequence position. If the gene was previously annotated, the species code and gene ID number are given. The transcript number is given for *C. sp. 33*. The gene start position and coding sequence length (CDS) are given.

| NSPF-1 Orthologs | | | |
| --- | --- | --- | --- |
| **Species** | **Orthologous Gene** | **Position** | **CDS (bp)** |
| *C. kamaaina* | CKA_174_0_13 | 174: 131,578 | 261 |
| *C. elegans* | *nspf-1* | II: 2,687,625 | 264 |
| *C. tropicalis* | CTP_g7984 | 629: 1,721,018 | 258 |
| *C. wallacei* | CWL_4_4_472 | 04: 4,472,618 | 258 |
| *C. sp. 33* | sp33_DN22189 | 309-52 | 258 |
| *C. latens* | CLA_103_0_109 | 130: 109,826 | 258 |
| *C. remanei* | CRE_3_5_858 | 3: 5,858,994 | 258 |
| *C. sinica* | CSN_7_0_121 | 00007: 121,864 | 255 |
| *C. briggsae* | CBG_05952 | IV: 7,468,227 | 255 |
| *C. nigoni* | CNG_IV_9_344 | IV: 9,344,857 | 255 |
| NSPF-3 Orthologs | | | |
| **Species** | **Orthologous Gene** | **Position** | **CDS (bp)** |
| *C. kamaaina* | CKA_174_0_12 | 174: 129,772 | 261 |
| *C. elegans* | *nspf-3* | II: 2,689,909 | 264 |
| *C. doughertyi* | CDG_455_30_9 | 00455: 30,964 | 261 |
| *C. tropicalis* | CTP_g7983 | 329: 1,720,080 | 258 |
| *C. wallacei* | CWL_4_4_473 | 04: 4,473,862 | 258 |
| *C. sp. 33* | sp33_DN3463 | 298-41 | 258 |
| *C. latens* | CLA_103_0_108 | 103: 108,660 | 258 |
| *C. remanei* | CRE_3_5_857 | 3: 5,857,810 | 255 |
| *C. sinica* | CSN_7_0_123 | 00007: 123,491 | 255 |
| *C. briggsae* | CBG_05951 | IV: 7,469,460 | 255 |
| *C. nigoni* | CNG_IV_9_343 | IV: 9,343,622 | 255 |
