# Supplementary material for: Proteomic and evolutionary analyses of sperm activation identify uncharacterized genes in Caenorhabditis nematodes
Source: BMC Genomics. 2018 Aug 7;19:593. doi: 10.1186/s12864-018-4980-7 (PMC6081950; doi:10.1186/s12864-018-4980-7)
Supplement: Supplementary file 4 — Consensus sequence alignments for the Nematode-Specific Peptide family, group D (NSPD). The amino acid sequence is largely conserved, except for the species-specific amino acid repeats in the middle of the gene. (PDF 79 kb) [file 12864_2018_4980_MOESM4_ESM.pdf]

Additional file 4. Consensus sequence alignments for the annotated Nematode-Specific Peptide family, group D (NSPD) orthologs in 11 *Caenorhabditis* species. Each species is represented by the consensus sequences for the 3-10 paralogs (90% identity threshold). Ambiguous amino acids are marked with an "X". *C. briggsae*, *C. sinica*, and *C. remanei* each had one gene with additional sequence on the N-terminus, which was excluded from the consensus. The region of species-specific repeats is between residues 39 and 51.

```
                1                  20                  40                  60               77
                |                  |                   |                   |                |
C.briggsae      MADKSAYMSAGGYXSGYMGSNASSSGYAXEDYASGGSGG----XXXNNNQGGSGGXXNPGXQVFKARTDQSCYLGP-
C.nigoni        MADKSAYMXAGGYSSGYMGXNASSSGYAREDYAXGGXGX-----XXXNNXXGSGGXXNPGXQVFKARTDQSCYLGP-
C.sinica        MADKSAYMXAGGYSSGYMGXNASSSGYAREXYAXGGXGXXASGGXNNNNXXGSGGXTNPGXQVFKARTDQSCYLGP-
C.latens        MADKSAYMSAGGXSSGYMGSNASSSGYAREDYASGGSGG----XXXNNQXQGSGGNTNPGXQVFKARTDQSCYLGP-
C.remanei       MADKSAYMSXGXYSSGYMGSNASSSGYAREDYASGGXGG---GXXNNXXQGGXGGNTNXGXQVFKARTDQSCYLGP-
C.sp33          MADKSAYMSAGGYXSGYMGSNASSSGYAREXYASGGSGG---XXXXNNXXXGXGGNXNPGXQVFKARTDQSCYLGP-
C.wallacei      MADKSAYMSAGGYSSGYMGSNASSSGYAREDYASGGSGG----GXXNNNNQGSGGNXNPGXQVFKARTDQSCYLGP-
C.tropicalis    MXDKXAYMSAGGYSSGYMGSNASSSGYXREDYASXGSGX----XXXNNXNQGSGXNXNPGXQVFKARTDQXCYLGP-
C.dougherti     MADKSAYMXAGGYXSGYMGXNASSSGYAREDYAXGGSGXX--XXXXNNXQGGXGGNTNPGXQVFKARTDQSCYLGP-
C.sp34          MADKSAYMXXGGYXSGYMGXNASSSGYAREXXAXGGXGX---------GNQGSGGXXXXGXQVFKARTDQSXYLGX-
C.elegans       MADKSAYMGAGGYGSGYMGSNASSSGYAREDYAQGQGGX---GGXXXXXXXGSGGNTNPGGQVFKARTDQSCYLGP-
C.kamaaina      MADKSAYMSAGGYSSGYMGSNASSSGYAREXYASGGSGX-------SGGXQGSGAVSNPGGQVFKARTDQSAYLGPS
```
