# Supplementary material for: Proteomic and evolutionary analyses of sperm activation identify uncharacterized genes in Caenorhabditis nematodes
Source: BMC Genomics. 2018 Aug 7;19:593. doi: 10.1186/s12864-018-4980-7 (PMC6081950; doi:10.1186/s12864-018-4980-7)
Supplement: Supplementary file 6 — Sequence alignments for the Nematode-Specific Peptide family, group F (NSPF) orthologous genes. Amino acid sequence is largely conserved across orthologs. (PDF 158 kb) [file 12864_2018_4980_MOESM6_ESM.pdf]

Additional file 6. Sequence alignments for the annotated Nematode-Specific Peptide family, group F (NSPF) orthologs in 11 *Caenorhabditis* species. Gene identifiers are comprised of the species code, scaffold or chromosome and the first three digits of the sequence position. If the gene was previously annotated, the species code and gene ID number are given. The transcript number is given for C. *sp. 33*. The predicted signal cleavage site is between residues 20 and 21.

NSPF-1

```
                1                  20                  40                  60                  80      89
                |                  |                   |                   |                   |        |
CBG_05952       MRF-FTIAL-FFCILSNVFAWYHGDRAALADDEYDPEAIENRQHIAKEYMAREKLRRRVREEIAKEEIRH-MYQREKIRKAMES-FNE-
CNG_IV_9_344    ...-....I-................V................Q..........................-.............-...-
CSN_7_0_121     ...-.F...-...........HT...V...........V....Q..........F...............-........R...D-.SD-
CRE_3_5_858     ...-.I...-...FI......HY...V...........VA...Q.....LI...F.T....Q.....L..-........R...D-..DN
CLA_103_0_109   ...-.I...-...F.......HY...V...........VA...Q.....LI...F.T....Q.....L..-........R...D-..DN
sp33_DN22189g1  ...-.I...-....I......NY...V...E........A...Q.....LI...F.T....Q.....L..-........R...D-..DN
CTP_g7984       ...-LI...-...FV......NY...V.......S...VA...Q.....LI...F.T..........L..-........R..DE-..DN
CWL_4_4_472     .K.-.I...-...V.......NY..SVG......N...VA..RQ..M..LA...F.T....Q..R..L..-..T..R..K..DQ-..DN
CE_nspf-1       ...-L.ITLAV....PTI..SHR.ERIEF.N........Y..HQLAR..IET.QA.SNI..DV....V..I.RL..-.KKNVEMY.GDR
CKA_174_0_13    .N.L.FV..-..VL....VA.NM..AIN.V.E....QAVM..RQLAR..K....F.A.............-QHV.AD......D-.GDY
```

NSPF-3

```
                1                  20                  40                  60                  80     88
                |                  |                   |                   |                   |       |
CBG_05951       MRFFT-IALFFCILSNV-FAWYHGDRVALADDEYDPEAIENRQQIAKEYMAREKFRRRIREEIAKEEIEHKYRREKIRRAMEA-FNE-
CNG_IV_9_343    .....-...........-................................................................S-...-
CSN_7_0_123     ....F-...........-...HT...............V...................V.........R...Q..........-.SD-
CRE_3_5_857     ....I-......FI...-...HY..QI..D....S...VA.........LI.....T....A.....L....Q.....K...E-..DM
CLA_103_0_108   ..L.L-......FI...-...HY..QI..D....S...VA..R......LI.....T....A.....L....Q.....K...D-..DM
sp33_DN3463g1   ....I-......FF..I-...HY.N.I..D....S...VS..R......LI.....T....A.....L....Q.....K...E-..DM
CTP_g7983       ...LV-F....FFVA.A-...NY...V..M...S....VA..R...M..L......T..........L....Q.....K...E-...M
CWL_4_4_473     .K..V-L......IA..-...SY...V..Y...SN...VA..R...M..L......TS...L..Q..L....Q.....KE..E-...M
CDG_455_30_9    .K..I-...........V...NY..SI.L....N....VA..E.............T.......R...R...K.....K..QT-...M
CE_nspf-3       .R.LFIT.V.....PTI-..RHR.ERIEF.T.......LY..H.L.R..IET.QA.SN...DV.....R.IM.LRE.KKNV.MY.GDR
CKA_174_0_12    .KLLILV.....L....-VSWNM..AI.LV.E.A..Q.VL..R.L.R..K...Q.RA.V.........R.Q.A.D...Q.INE-..DY
```
